# MicroRNA roles in beta-catenin pathway

**DOI:** 10.1186/1476-4598-9-252

**Published:** 2010-09-21

**Authors:** Kai Huang, Jun-Xia Zhang, Lei Han, Yong-Ping You, Tao Jiang, Pei-Yu Pu, Chun-Sheng Kang

**Affiliations:** 1 Department of Neurosurgery, Tianjin Medical University General Hospital, Tianjin 300052, China; 2 Laboratory of Neuro-Oncology, Tianjin Neurological Institute, Tianjin 300052, China; 3 Key Laboratory of Neurotrauma, Variation and Regeneration, Ministry of Education and Tianjin Municipal Government, Tianjin 300052, China; 4 Department of Neurosurgery, The First Affiliated Hospital of Nanjing Medical University, Nanjing 210029, China; 5 Department of Neurosurgery, Tiantan Hospital, Capital Medical University, Beijing 100050, China

## Abstract

β-catenin, a key factor in the Wnt signaling pathway, has essential functions in the regulation of cell growth and differentiation. Aberrant β-catenin signaling has been linked to various disease pathologies, including an important role in tumorigenesis. Here, we review the regulation of the Wnt signaling pathway as it relates to β-catenin signaling in tumorigenesis, with particular focus on the role of microRNAs. Finally, we discuss the potential of β-catenin targeted therapeutics for cancer treatment.

## Introduction

Altered function of components of the canonical Wnt/β-catenin signaling pathway is associated with cancer, as multiple Wnt/β-catenin target genes are regulators of cell proliferation, metastatic potential and tumorigenesis [1,2]. In the absence of Wnt, cytoplasmic β-catenin dissociated from the E-cadherin/β-catenin/α-catenin complex [3] is rapidly phosphorylated by activated glycogen synthase kinase 3β (GSK3β) at Ser33, Ser37, and Thr41[4] and phosphorylated by casein kinase Iα (CK Iα) at Ser45 [5]. These phophorylations prevent the nuclear accumulation of β-catenin, leading to its ubiquitination and subsequent degradation by the ubiquitin/proteasome system [6,7]. Upon binding of Wnt to the transmembrane receptor Frizzled (FZD), in complex with co-receptors Low-density-lipoprotein receptor-related proteins 5 and 6 (LRP5/6) [8], the Wnt-FZD-LRP-5/6 complex phosphorylates and activates Disheveled (Dsh) [9]. Dsh activation inhibits GSK3β, subsequently decreases β-catenin degradation by the ubiquitination and proteasomal pathways. In turn, β-catenin accumulates in the cytoplasm and nucleus, where it interacts with coregulators of transcription including T cell factor/lymphocyte enhancer factor (Tcf/Lef) to form a β-catenin/Lef/Tcf complex [10]. This complex regulates transcription of multiple genes involved in cellular proliferation, differentiation, survival and apoptosis, including c-myc and cyclin D [11,12]. Recent reports suggest that nuclear GSK3β can additionally inhibit β-catenin transcription indirectly, via binding and phosphorylation of Axin and then reducing the transcriptional activity of the β-catenin/Tcf/Lef complex [13]. Hyperactivation of β-catenin caused by the overexpression of Wnt or mutation of CTNNB1 (the gene which encodes β-catenin), GSK3β, Axin or APC is a common cause of carcinoma [14,15]. Specifically, the mutation of APC is a leading cause of colorectal carcinomas [16], and the relative mRNA and protein expression of β-catenin positively correlates with histological malignancy in astrocytoma [17,18].

**Figure 1:**
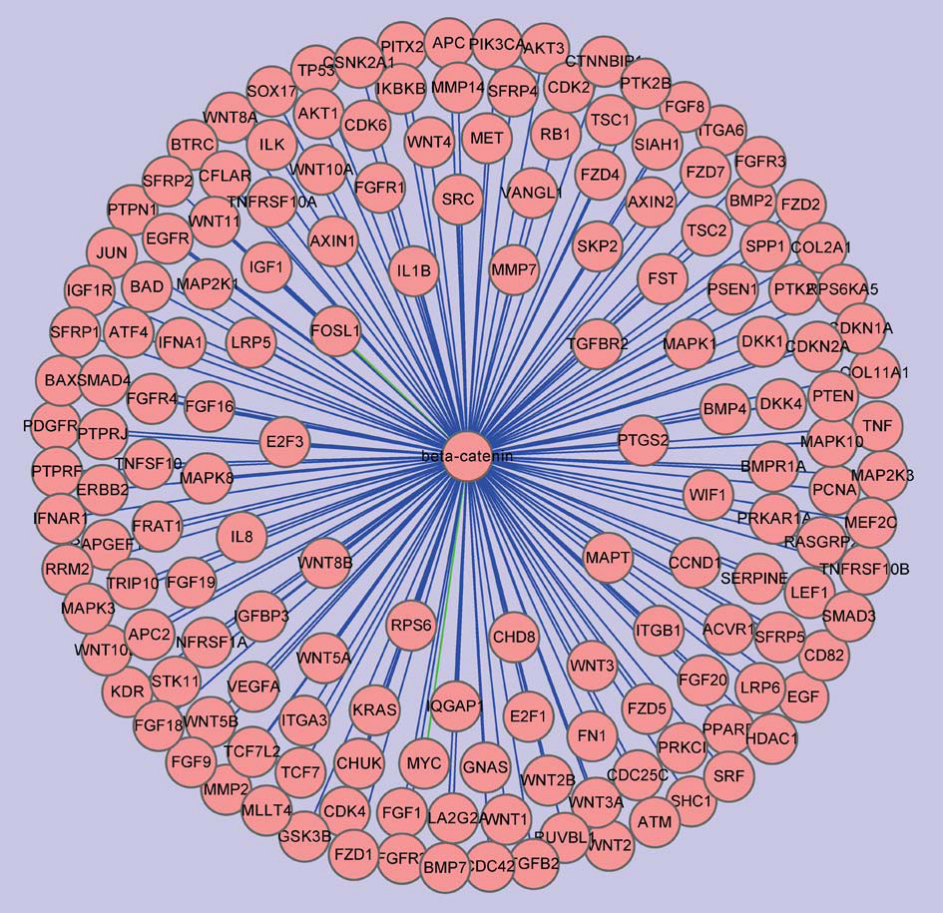
**Visualization of β-catenin interaction network**. 10018 articles describing putative interactions between β-catenin and other genes were identified through querying Pubmed with: (catenin or CTNNB or CTNNB1) and ("1980/01/01"[PDAT]: "2009/05/24"[PDAT]) and text mining. 213 genes (including Tcf4 and Lef, ect.) formed a complex with β-catenin (interaction relations is associate, bind, etc.) and β-catenin interaction network was constructed by Cytoscape.

**Figure 2:**
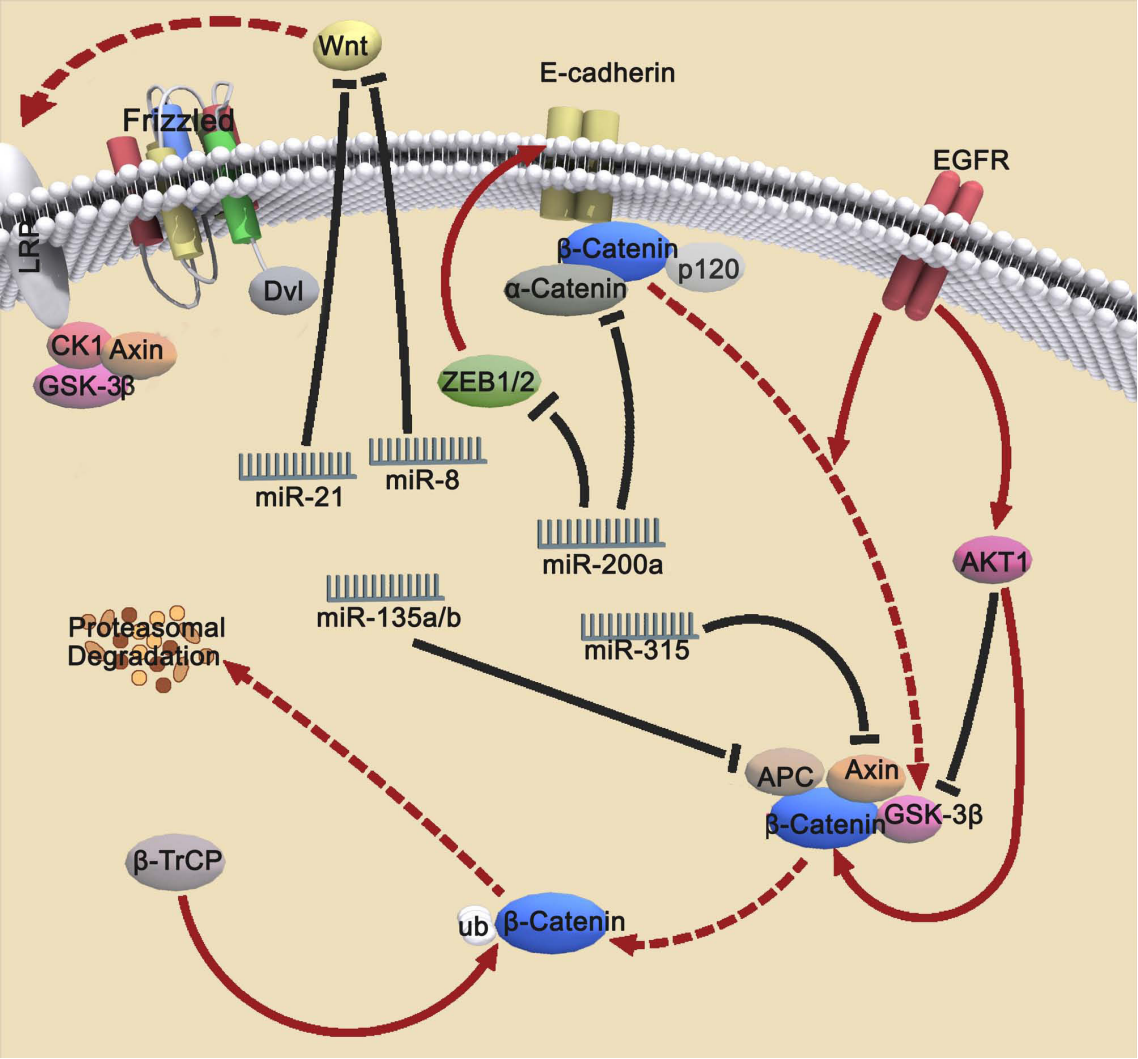
**Upstream regulators of β-catenin transcriptional activity**. For details see the text. EGFR, AKT1, miR-315 and miR-135a/b upregulate β-catenin transcriptional activity, whereas miR-200a, miR-21 and miR-8 downregulate β-catenin transcriptional activity.

**Figure 3:**
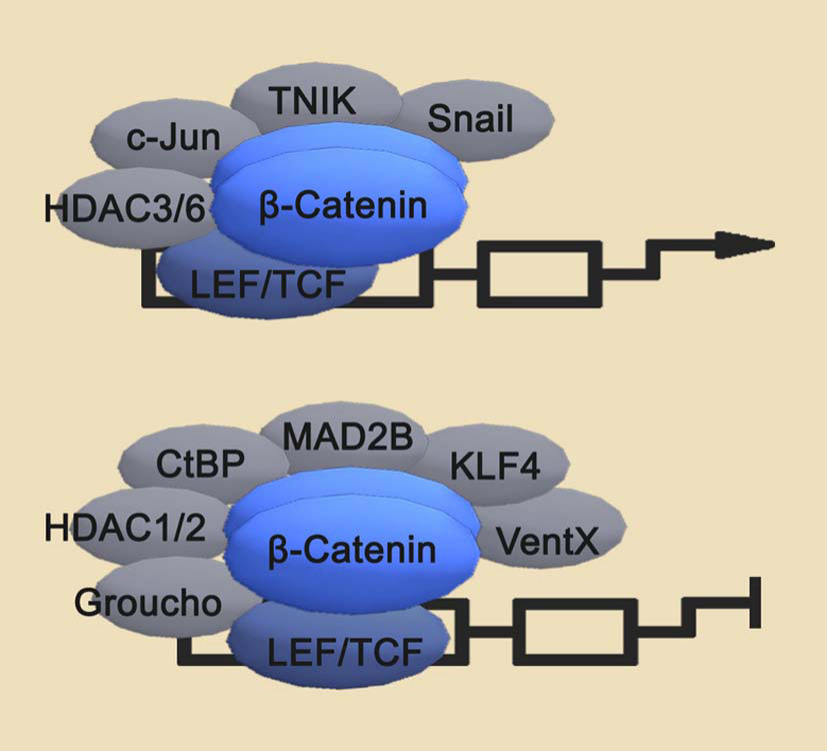
**Coregulators of β-catenin transcriptional ativity**. Via interacting with the β-catenin/Tcf/Lef complex, HDAC3/6, c-Jun, TNIK and Snail upregulate β-catenin transcriptional activity (up), whereas HDAC1/2, CtBP, Groucho, KLF4, MAD2B and VentX downregulate β-catenin transcriptional activity (down).

Up to now, there have been more than ten thousands of β-catenin related publications in MEDLINE (Pubmed with: beta catenin). Over the last 10 years, the number of new entries about β-catenin in MEDLINE has grown at a 9.3% compounded annual growth rate, and the number of new entries in MEDLINE each year has grown at a compounded annual growth rate of 3.1% [19]. Furthermore, we queried Pubmed with: (catenin or CTNNB or CTNNB1) and ("1980/01/01"[PDAT]: "2009/05/24"[PDAT]), and identified 10018 articles describing putative interactions between β-catenin and other genes (543 genes) by text mining. 213 genes (including Tcf4 and Lef, ect.) interact with β-catenin (interaction relations is associate, bind, etc.) and a β-catenin interaction network was constructed (Fig 1). Therefore, great progress in biological function and molecular mechanism of β-catenin has been made, and new highlights for β-catenin pathway are deserved to summary. In this review, we focus on modulators of the Wnt/β-catenin signaling pathway, describing new findings of upstream regulators (Fig. 2), coregulators (Fig. 3) and downstream targets, with special focus on the function of microRNAs (Table 1). Finally, we aim to emphasize the importance of the Wnt/β-catenin signaling pathway in cancer, describing β-catenin-targeted reagents that hold promise as chemotherapeutics.

**Table 1:** Novel modulators of Wnt/β-catenin signaling pathway

upstream regulators	function	coregulators	function	downstream targets	transcription
EGFR [20,21]	↑	CtBP [53]	↓	AKT1 [77]	→
AKT1 [26,28]	↑	Groucho [52]	↓	STAT3 [86,87]	→
miR-135a/b [50]	↑	HDAC1/2 [61,62]	↓	Gbx2 [82]	→
miR-315 [51]	↑	KLF4 [72]	↓	MMP1 [20]	→
JNK [34,35]	↑ or ↓	MAD2B [73]	↓	Foxc1 [84]	→
miR-200a [36,42]	↓	VentX [74]	↓	StarD7 [85]	→
miR-21 [43]	↓	HDAC3/6 [64,65]	↑	E2F1 [87]	←
miR-203 [48]	↓	c-Jun [66]	↑	p16INK4a [83]	←
miR-8 [49]	↓	TNIK [70]	↑	miR-15/16 [93]	←
		Snail [71]	↑	miR-122a [94]	←
				miR-375 [95]	←

↑: Regulator upregulates β-catenin transcriptional activity

↓: Regulator downregulates β-catenin transcriptional activity

→: Target transcription is upregulated by activated β-catenin signaling

←: Target transcription is downregulated by activated β-catenin signaling

### Upstream Regulators of β-catenin transcriptional activity

#### EGFR activation phosphorylates β-catenin at Tyr654

Activation of EGFR induces phosphorylation and activation of CK2α via ERK. CK2α activation signals phosphorylation of α-catenin at S641, and triggers loss of α-catenin binding to β-catenin and subsequent activates β-catenin/Lef/Tcf transcriptional activity [20]. Additionally, EGFR activation leads to phosphorylation of β-catenin on Tyr654 residue. EGFR binds to β-catenin and induces its tyrosine phosphorylation [21], definitive evidence that EGFR directly phosphorylates β-catenin at Tyr654 remains elusive. However, Tyr654 phosphorylation results in dissociation of the E-cadherin/α-catenin/β-catenin complex [22]. Further, EGFR regulates β-catenin localization and stability, transcriptional activity, and tumor progression in oral cancer [23]. In addition, phosphorylation of β-catenin at Tyr654 and 670 mediated by interaction of the hepatocyte growth factor (HGF) with its cognate receptor (Met), regulates its nuclear translocation and activation [24]. Whether the Tyr654 residue of β-catenin is a direct target of EGFR requires further determination.

#### β-catenin is directly phosphorylated at Ser552 by AKT1

AKT1, a serine/threonin kinase also known as PKB, functions as a key mediator of the PI3K/AKT pathway. Activated AKT1 is phosphorylated at Thr308 and Ser473, thereby regulating β-catenin by inducing phosphorylation of effector molecules GSK3β, mTOR and BAD [25-27]. Specifically, GSK3β phosphorylation at Ser9 by AKT1 inhibits the N-terminal phosphorylation of β-catenin [26]. A resent study, however, suggests that AKT1 can regulate β-catenin directly by inducing phosphorylation at Ser552 in vitro and in vivo [28]. This Ser552 residue was confirmed as a phosphorylation site by liquid chromatography-coupled ion trap mass spectrometry (LC-MS/MS), and validated by site directed mutagenesis. Ser552 phosphorylation results in β-catenin translocation from the cytosol into the nucleus, increasing Tcf4/Lef1 transcriptional activity and promoting tumor cell invasion. β-catenin phosphorylation of Ser552 was additionally identified in intestinal crypts [29]. Cells exhibiting nuclear β-catenin phosphorylation at Ser552 were frequently clustered together and found at sites of crypt fission and invagination during crypt budding, revealing an interesting role for AKT1 in stem cell biology. In sum, the phosphorylation of β-catenin at Ser552 by AKT1 increases nuclear translocation and retention, enhancing Tcf4/Lef1 complex transcriptional activity to promote tumor cell invasion and stem cell migration. The exact mechanism how the phosphorylation of β-catenin at Ser552 positively regulates the expression of its target genes remains unclear. One possibility exists that phosphorylation of Ser552 may modulate the translocation of β-catenin through nuclear pores, while a second possibility remains that phosphorylation by AKT1 occurs within the nucleus and affects function rather than localization [29]. Further investigation is needed to determine whether the phosphorylation of β-catenin at Ser552 exerts the same effect as stabilized β-catenin on binding to the Tcf4/Lef1 complex and activating overexpression of Wnt target genes.

#### β-catenin is phosphorylated by JNK

The c-Jun N-terminal kinase (JNK) is a stress-activated protein kinase that is a member of an evolutionarily conserved sub-family of the mitogen-activated protein kinase (MAPK) family of serine/threonine protein kinases. In early Xenopus embryos, high level of nuclear JNK negatively regulates the canonical Wnt/β-catenin signaling pathway by expelling β-catenin from the nucleus [30]. A recent study demonstrates that JNK directly binds to and phosphorylates β-catenin at Ser37 and Thr41, regulating the formation of adherens junctions in human epithelial cell lines [31]. Pharmacologic inhibition of JNK with SP600125 or by expression of dominant negative JNK (JNKDN) decreased the phosphorylation of β-catenin and induced translocation of both β-catenin and E-cadherin to the cell surface. This event disrupts cell-cell adhesion stimulated by okadaic acid (OA) treatment, suggesting that cell adhesion is dynamically regulated by JNK. Further, data suggests that JNK regulation of cell adhesion may contribute to processes including wound healing, tumor proliferation and metastasis.

Loss of JNK 1/2 results in increased expression of β-catenin, and β-catenin/Tcf complex target genes, including c-myc [31,32]. Phosphorylation and degradation of β-catenin by JNK 1/2 in these studies was blocked by pharmacologic inhibition or RNAi knockdown of GSK3β. Similarly, immunoblotting revealed that JNK1/2 activated GSK3β activation, promoting β-catenin degradation. The interaction between JNK1/2, β-catenin and GSK3β, confirmed by coimmunoprecipitation and confocal microscopy, suggests that β-catenin is phophorylated and degraded by JNK/β-catenin/GSK3β complex. Studies by Ximei Wu and colleagues demonstrated that JNK2 is more potent than JNK1 in phosphorylating β-catenin at Ser191 and Ser605 in ST2 cells [33]. In contrast to the phosphorylations at Ser33, Ser37 and Thr41 by GSK3β in canonical Wnt signaling pathway, the phosphorylations at Ser191 and Ser605 of β-catenin results in stabilization of the protein, translocation to the nucleus, and increased bind of and transcription by the Tcf/Lef complex. Further, JNK enhances the transcriptional activity of β-catenin by phophorylating and activating c-Jun/AP-1, a well characterized coactivator of the β-catenin/Tcf/Lef complex [34]. However, a recent report has revealed that phosphorylation of JNK1 induced by selenium suppresses β-catenin in vivo, resulting in cell growth inhibition [35]. Thus, the discrepancy in the regulation of β-catenin/Tcf complex transcriptional activity by JNK may vary depending on cell type and microenvironment.

#### Targeting β-catenin-mediated transcription by miR-200a

MicroRNAs are single-stranded noncoding RNAs of 21 to 23 nucleotides in length that repress translation or induce cleavage of target mRNAs that they are partially complementary to at the 3'or 5' untranslated region (UTR). MiR-200a was recently reported to downregulate β-catenin-mediated transcription via two different mechanisms. MiR-200a targets the mRNA of the E-cadherin repressor proteins ZEB1(also known as Tcf8) and ZEB2 (also known as SIP1), subsequently increases the total E-cadherin available for binding to β-catenin and induces formation of the cell-cell adhesion complex [36]. β-catenin is subsequently phosphorylated at Ser33, Ser 37, Thr 41 and Ser45 by the tumor destruction complex and degraded by the ubiquitin/proteasome system. Reduction of miRNA-200a upregulates free cytoplasmic and nuclear β-catenin levels and then induces epithelial to mesenchymal transition (EMT), revealing a role for β-catenin in this process [37-41]. A second, novel mechanism for downregulation of β-catenin activity by miR-200a was recently proposed in meningiomas [42]. MiR-200a, down-regulated in most meningiomas, regulates expression of β-catenin and activation of Wnt/β-catenin signaling via directly targeting the 3'UTR of β-catenin mRNA. While miR-200b and miR-200c, two additional members of miR-200 family, show no impact on β-catenin expression.

#### MiR-21 targets WNT1 gene expression

Protein and mRNA analyses identified that WNT1 is translationally repressed by miR-21. Antagonism of the effects of miR-21, either by transfection with miRNA inhibitors or by exogenous addition of Wnt-1, inhibits human monocyte-derived dendritic cell (MDDC) differentiation, suggesting that miR-21 has a key regulatory role in MDDC differentiation [43]. Interestingly, miR-21 was previously shown to additionally target the tumor suppressor PTEN, RECK and PDCD4 and induce tumorgenesis [44-47], suggesting a general role for miR-21 in tumor progression.

#### MiR-203 directly targets Lef1

MiR-203 directly targets the Wnt signaling transcription factor Lef1 in zebrafish [48]. The 3'UTR of Lef1 contains two potential miRNA recognition elements (MREs) for miR-203. Expression of Lef1 from mRNAs lacking 3'UTR recognition elements can rescue the effects of excess miR-203, demonstrating that these effects are due to specific regulation of Lef1 by miR-203. MiR-203 was found to be significantly downregulated during fin regeneration. Further, repression of Lef1 by miR-203 blocks fin regeneration, whereas loss of miR-203 results in excess Lef1 levels and fin overgrowth.

#### *MiR-8 impacts Wnt/*β-catenin signaling via the *Wg pathway*

While it is known that mammalian miR-8 family members promote adipogenesis, possibly by inhibiting Wg signaling, the mechanism of this event remains elusive. Three potential mechanisms have been reported. Expression of miR-8 may potently antagonize Wg signaling by directly binding the 3'UTR of wntless (wls) and inhibiting Wg signaling in part by preventing Wg secretion [49]. MiR-8 additionally may impact downstream of the Wg signal, by repressing Tcf protein levels. While miR-8 does not directly target the two putative 3' UTRs of Tcf mRNA, it may directly target the Tcf mRNA independently of its 3'UTR or through an indirect mechanism. Finally, miR-8 may exert its impact by directly targeting CG32767, a positive regulator of the Wg pathway.

#### MiRNAs target APC/Axin

MiR-135a and miR-135b target the 3'UTR of APC and suppress its expression, subsequently increasing Wnt signaling by stabilizing β-catenin [50]. Similar to the correlation of loss-of-function APC to colorectal tumorigenesis, increasing expression of miR-135a and miR-135b promoted progression of colorectal adenomas to adenocarcinomas. This association is independent of the status of APC mutation or promoter hypermethylation in the tumors. Further, MiR-315 directly targets Axin and Notum, two negative regulators of Wg signaling, in Drosophila cells mice, resulting in Wnt pathway activation [51].

### Coregulators of β-catenin transcriptional ativity

#### Coregulation of the β-catenin/Tcf4/Lef1 complex

The Tcf family consists of four members in vertebrates, including Tcf1, Tcf3, Tcf4 and Lef1. Each member contains a DNA-binding high mobility group (HMG) box, and a highly conserved β-catenin interacting region. In the absence of β-catenin, Tcf4 recruits co-repressors HDAC1, CtBP, and Groucho/transducin-like enhancer of Split (TLE) to silence expression of target genes [52-54]. Groucho/TLE proteins repress the basal transcriptional machinery [55] as well as recruit HDACs, which contribute to corepression by direct action on chromatin [56]. A direct interaction between Groucho and Lef1 occurs via a small region in the context regulatory domain (aa237-256) and a region in the highly conserved HMG DNA binding domain (aa296-396) [52]. However, as β-catenin accumulates in the nucleus, Groucho/TLE is displaced from Tcf/Lef by β-catenin binding to C-terminal of Lef1 (residure252-397). This C-terminal DNA-binding domain overlaps with the Groucho/TLE binding site [57]. β-catenin then recruits co-activators through its N-terminal and C-terminal transactivation domains, including BCL9/Legless [58,59] and p300/CBP [60].

#### β-catenin transcriptional activity is regulated by Histone deacetylases

Histone deacetylases (HDACs) are a class of enzymes that remove acetyl groups from ε-N-acetyl lysine amino acids on a histone. HDACs attenuate β-catenin transcriptional activity by binding to Lef, forming a HDAC-Lef complex that hypoacetylates the promoter region of β-catenin/Tcf/Lef complex target genes [52,61]. Upon β-catenin recruitment and accumulation in the nucleus, however, HDACs dissociate from Lef and form a HDAC/β-catenin complex, attenuating the enzymatic activity of HDAC and allowing residual β-catenin to bind to Lef and recover the transcriptional activity of the β-catenin/Tcf/Lef complex [61]. Recently, HDAC1/2 were demonstrated to regulate oligodendrocyte differentiation, at least in part, via disruption of β-catenin/Tcf interactions [62]. HDAC1/2 competes with β-catenin for Tcf4 interaction, thus regulating β-catenin/Tcf4 complex transcriptional activity. Specifically, HDAC1/2 regulate expression of differentiation inhibitors2/4 (ID2/4), target genes of the β-catenin/Tcf4 complex that negatively regulate oligodendrocyte differentiate. The displacement of Tcf4 from β-catenin by HDAC1/2 or the formation of ternary complex HDAC/β-catenin/Tcf4 switches off transcription of ID2/4. Thus, transcriptional co-repressors HDAC1 and HDAC2 compete with β-catenin for Tcf4 interaction to promote oligodendrocyte differentiation in a manner, at least in part, dependent on the expression of β-catenin/Tcf4 complex target genes ID2/4. Similarly, in the zebrafish retina, HDAC1 antagonizes Wnt signaling to suppress both cell-cycle progression and subsequent inhibition of neurogenesis [63].

Recent evidence identifies that HDAC3 expression may enhance transcription activity of the Wnt/β-catenin pathway [64]. Stable HDAC3 knockdown attenuated activation of the Wnt pathway by increasing plasma membrane localization and reducing nuclear accumulation of β-catenin. Further, knockdown of HDAC3 induced increased expression of TLE1/4, which provided competition with β-catenin for interaction with Tcf4/Lef1, thereby antagonizing transcription. Moreover, HDAC6 may similarly enhance transcription activity of the Wnt/β-catenin pathway. HDAC6 deacetylates β-catenin at lysine 49 and inhibits its phosphorylation at Ser45, resulting in its nuclear translocation and accumulation [65].

#### c-Jun functions as a coactivator of the β-catenin/Tcf complex

c-Jun is well known as a downstream effector of the β-catenin/Tcf complex [66]. c-Jun heterodimerizes and forms a functional transcription factor complex termed AP-1 in combination with c-Fos. Recently, c-Jun and AP-1 have been demonstrated by genome wide ChIP-on-chip analysis to interact with β-catenin and form a c-Jun/AP-1/β-catenin/Tcf complex, which prevents β-catenin phosphorylation and degradation and increases its activity [67]. A recent study additionally proved that c-Jun directly binds to the β-catenin/Tcf complex [68], suggesting that c-Jun functions as an adaptor protein to mediate the association of Dsh with the β-catenin/Tcf complex on the promoter of Wnt target genes. The Dsh/c-Jun/β-catenin/Tcf complex similarly increases β-catenin stabilization and positively regulates the activity of Wnt signaling pathway. Each of these functions of c-Jun is dependent on its phosphorylation by JNK. In contrast, Wnt3a-induced Tcf reporter activity is not affected by knockdown of endogenous c-Fos, indicating that c-Fos does not impact canonical Wnt signaling as c-Jun [69]. Whether c-Fos binds to β-catenin or the c-Fos/β/catenin/Tcf complex is an avenue for further investigation.

#### Additional coregulators of the β-catenin/Tcf/Lef complex

Multiple novel upstream regulators of β-catenin/Tcf/Lef complex transcriptional activity have been identified. Positive modulators of β-catenin activity include Nck-interacting kinase (TNIK) and Snail. TNIK expression is enriched in the nuclei of Wnt-activated intestinal crypts, but not cells of the villus, where it is specifically recruited to the promoters of Wnt target genes Axin2 and c-Myc [70]. In vitro immunoprecipitation and kinase assays reveal that TNIK directly binds to both Tcf4 and β-catenin in these cells, phosphorylating and activating the transcription activity of Tcf4. Snail interacts with β-catenin at its N-terminus and increases its transcriptional activity independent of Tcf4. Snail, which is transcriptionally repressed by GSK3β, up-regulates TGF-3β gene expression through β-catenin/Tcf4 and promotes EMT [71]. In contrast, multiple negative regulators of β-catenin signaling exist as well. Krüppel-like factor 4 (KLF4), a transcription factor highly expressed in normal human intestine and critical for intestinal differentiation, inhibits Wnt signaling by direct interaction with the C-terminal transactivation domain of β-catenin, blocking recruitment of p300/CBP to this domain. KLF4 inhibition of p300/CBP recruitment results in inhibition of both β-catenin acetylation as well as histone acetylation of Wnt target genes. KLF4 additionally directly interacts with Tcf4 independent of β-catenin [72]. MAD2B, a novel Tcf4 coregulator identified by coimmunoprecipitation, downregulates β-catenin/Tcf/Lef complex activity by interrupting the DNA binding ability of Tcf4 [73]. Finally, VentX, a human Xom homologue, is a Lef/Tcf-associated inhibitor of canonical Wnt/β-catenin signaling and a negative regulator of cell proliferation[74].

### Downstream targets of β-catenin

**Figure 4:**
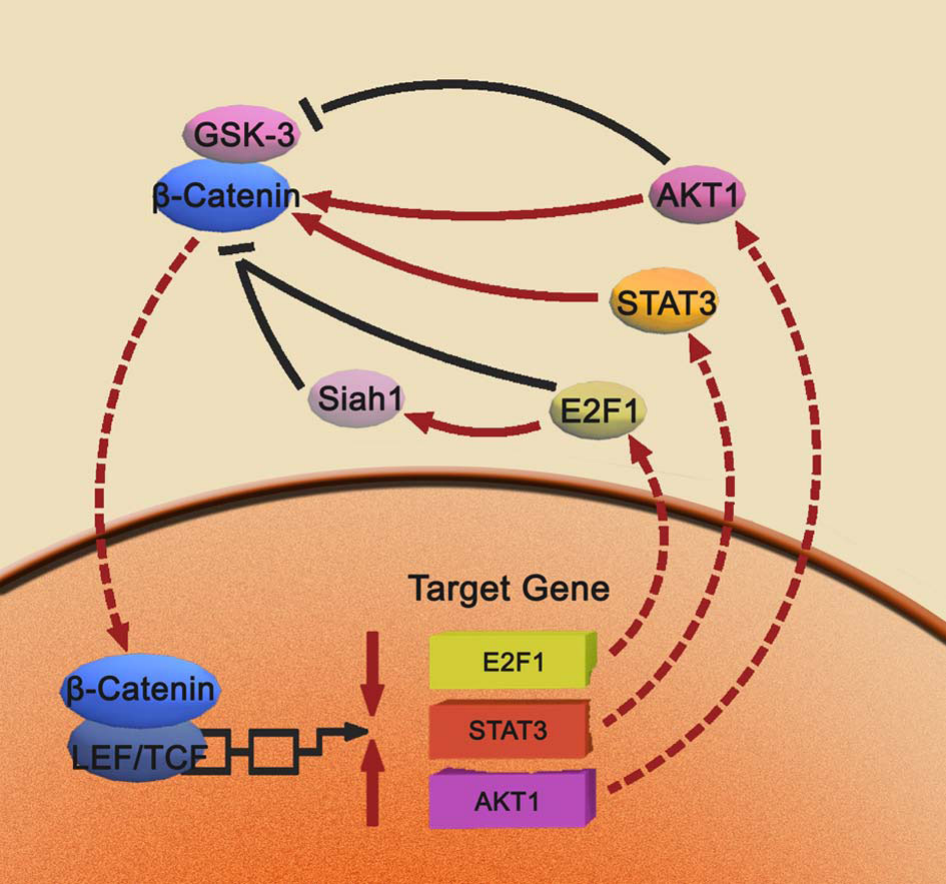
**Positive feedback loops between β-catenin and downstream targets**. The β-catenin/Tcf complex directly binds to binding element site (TBE) of the AKT1 and STAT3 gene promoter. In turn AKT1 activation induces β-catenin phosphorylation at Ser552 directly or by GSK-3β phosphorylation and STAT3 activation increases the nuclear accumulation of β-catenin, thereby increasing β-catenin nuclear translocation and enhancing transcriptional activity. In addition, the E2F1 promoter activity is repressed by overexpression of β-catenin/Tcf, and E2F1 inhibites β-catenin transcription directly or via upregulation of Siah1.

Upon translocation to the nucleus, β-catenin interacts with Tcf4 and Lef1 to regulate a wide range of gene expression at the transcriptional level [75]. The complex binds to A-C/G-A/T-T-C-A-A-A-G motifs, an evolutionarily conserved consensus motif on the promoter of target genes [76]. While c-myc, cyclin D and c-Jun expression are well-characterized as regulated by this complex [11,12,66], multiple new targets have recently been identified http://www.stanford.edu/~rnusse/pathways/targets.html. Here, we summarize the novel downstream targets of β-catenin and the positive feedback loops between β-catenin and the downstream targets (Fig 4).

#### AKT1 is regulated by β-catenin at the transcriptional level

Several non-steroidal anti-inflammatory drugs (NSAIDs) suppress β-catenin expression in human cancer cell lines, including aspirin, indomethacin, sulindac and etodolac [77-79]. Nitric oxide-donating aspirin (NO-ASA) inhibits the transcriptional activity of β-catenin/Tcf far more potently than aspirin, and inhibits the growth of colorectal cancer cells (CRC) more efficiently [80]. Aspirin and indomethacin downregulate β-catenin activity through increasing the stabilization of phosphorylated β-catenin in time- and concentration-dependent manners. These studies identified that aspirin induces a decrease in expression of AKT1, which is regulated by the β-catenin/Tcf complex as revealed by reporter assay [77]. Functionally, active AKT1 induced by β-catenin decreases Bax activation, oligomerization, and translocation to mitochondria, thus antagonizing mitochondrial injury and apoptosis [81]. As AKT2 and AKT3 are closely related and highly conserved homologs of AKT1, whether AKT2 and AKT3 are targets of Wnt/β-catenin signaling will be subject to additional studies.

#### Determination of novel β-catenin-regulated genes by ChIP assay

ChIP and transgenic analysis identified that the Gbx2 regulatory elements that drive expression in the neural crest (NC) respond directly to Wnt/β-catenin signaling. Loss-of-function experiments using antisense morpholinos against Gbx2 inhibit NC protein expression and expand the preplacodal domain, whereas Gbx2 overexpression leads to transformation of the preplacodal domain into NC cells. Previous studies identified a region of 500 bp upstream of Gbx2 that contains three (1-3) Lef/Tcf consensus sequences, termed the Gbx2 enhancer [82]. Similarly, the β-catenin/Tcf4 complex binds to a specific site on matrix metalloproteinase 1 (MMP1) promoter and governs MMP1 gene and protein expression, regulating cell migration in collagen and gelatin [20]. Further, β-catenin directly represses p16INK4a expression by binding to its promoter. Activated β-catenin directly represses the expression of p16INK4a through an evolutionarily conserved Lef/Tcf site in its promoter [83]. ChIP analysis additioanlly determined that β-catenin bound to conserved regions of mouse genomic DNA proximal to the Foxc1 transcriptional start site, revealing that Foxc1 is a direct target of β-catenin [84]. Despite β-catenin regulation of Foxc2 expression, no transcriptional start site has yet been identified. Finally, ChIP analysis revealed that β-catenin and Tcf4 activated the human StarD7 gene interacting with its promoter region [85].

Previously, WNT/β-catenin was suggested to regulate STAT3 at the mRNA and protein level, suggesting that STAT3 maybe a direct target of β-catenin [86]. Further investigation was performed by using EMSA and ChIP assay, confirming that the β-catenin/Tcf4 complex directly bound to the Tcf4 binding element site (TBE) of the STAT3 gene promoter [87]. These data confirm that STAT3 is regulated by β-catenin in the transcriptional level. Of note, STAT3 activation increases the nuclear accumulation of β-catenin, leading to a positive feedback loop between β-catenin and STAT3 [88]. Similarly, the E2F1 promoter was found to contain two putative Tcf-binding elements, and promoter activity is inhibited by overexpression of β-catenin/Tcf [89]. This event represents a positive feedback loop for β-catenin transcriptional activity, as E2F1 represses β-catenin transcription directly [90] or via upregulation of Siah1 [91]. Additionally, Lef1 activates E2F1 by attenuating the interaction between E2F1 and HDAC1 in a β-catenin-independent manner [92].

#### β-catenin downregulates miR-15, miR-16, miR-122a and miR-375 expression

Wnt/β-catenin signaling regulates miR-15/16 maturation rather than its transcription, as overexpression of β-catenin inhibits the expression of mature miR-15 and miR-16 isoforms. The mechanism of Wnt control of miR-15 and miR-16 maturation is unknown, but perhaps works through a protein complex controlled by or containing β-catenin. β-catenin control of the intensity and spatial pattern of Nodal responsiveness is thought to be regulated by miR-15 and miR-16 expression [93]. Upregulation of miR-122a expression in mutant APC cells induces a gain of wild type APC function, indicating that miR-122a works downstream of APC and suggests that miR-122a expression is lost or downregulated in APC-driven gastrointestinal cancers. Further, restoration of miR-122a expression significantly suppressed migration, invasion, anchorage-independent growth, and in vivo tumorigenicity of hepatocellular carcinoma cells. The mechanism by which miR-122a expression bypasses loss-of-function APC is unknown. Data suggests that miR-122a may be a novel target of APC/β-catenin signaling pathway, and that down-regulation of miR-122a mediated by aberrant APC/β-catenin signaling is important to the pathogenesis of gastrointestinal cancers [94]. Additionally, miR-375 has been demonstrated to be downregulated by β-catenin [95]. The function of miR-375 and the transcriptional mechanism that miR-375 regulated by β-catenin are not clear and for further investigation.

### Anti-β-catenin agents

The β-catenin complex plays a critical role in tumorgenesis, angiogenesis, and progression of metastasis, making it an attractive therapeutic target for chemotherapy. Current drugs that target β-catenin include Quercetin, which inhibits the transcriptional activity of β-catenin by disrupting the binding of β-catenin and Tcf4 and suppressing their translocation to the nucleus [96,97]. Similarly, Aspirin was demonstrated to increase phosphorylation of β-catenin but not decrease its nuclear translocation [77]. Neither quercetin nor aspirin, however, attenuate the protein level of total β-catenin. PKF118-310, PKF115-584, and CGP049090 reduce the binding of β-catenin and Tcf4, resulting in induction of G1/S phase arrest, inhibition of cell growth and activation of apoptosis [98]. 2,4-diaminoquinazolines and their analogues, a novel series of β-catenin antagonist, as well as multiple antagonists not presented here, have been demonstrated to inhibit the growth of colorectal cancer [99,100], proving the value of β-catenin antagonists as potential therapeutics. Taken together, we believe that the potential for β-catenin antagonists as potent chemotherapeutics for Wnt-driven malignancies is great, and further study of these and related agents will yield effective therapies for human cancer.

## Conclusion and Perspectives

**Figure 5:**
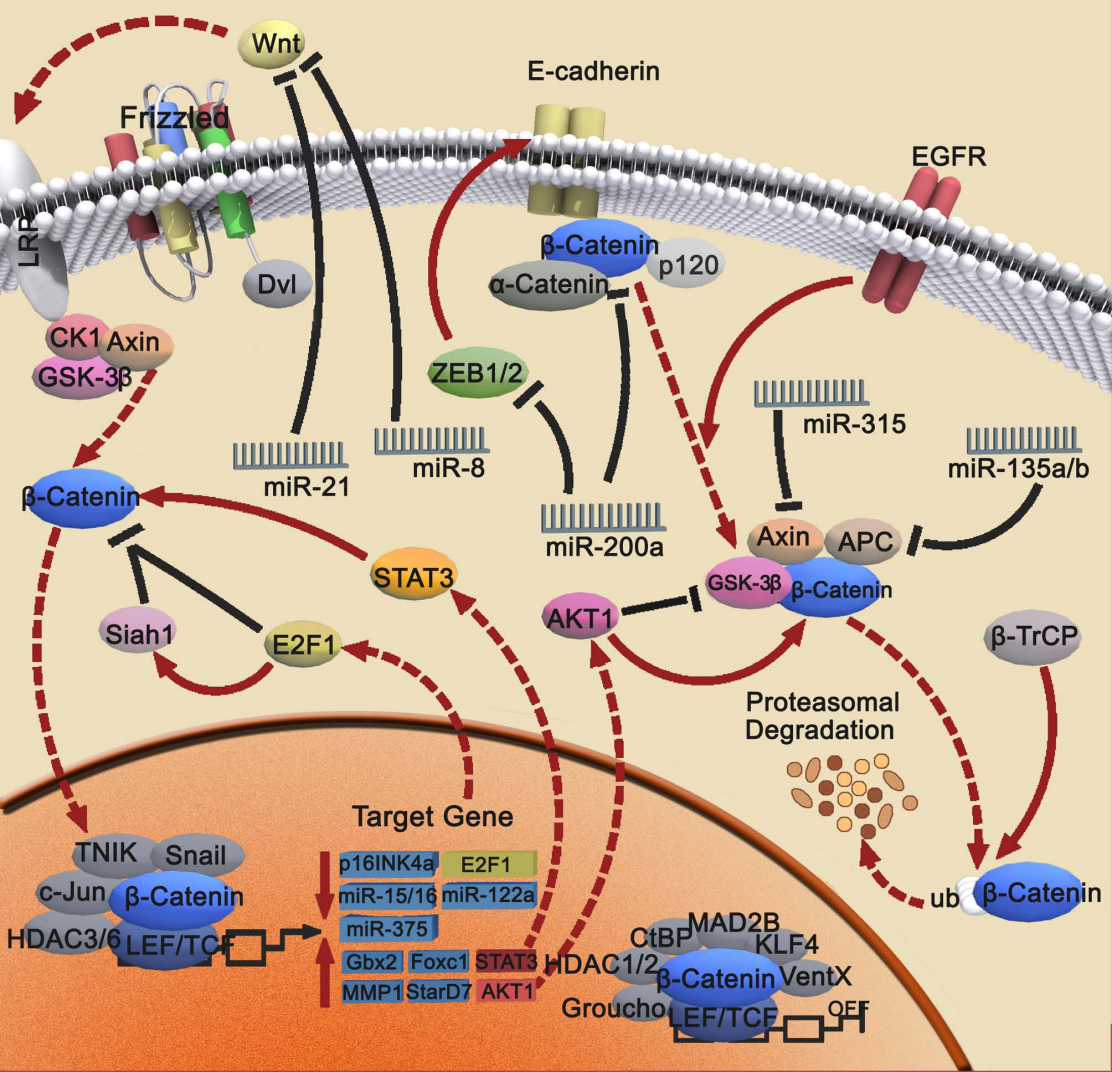
**Novel molecular regulation of Wnt/β-catenin pathway**. Particular Wnt/β-catenin signaling pathways affected by upstream regulators, coregulators and downstream targets are described in detail in the review.

Although Wnt/β-catenin transcriptional activity has been studied over the past several years, molecular regulation of Wnt/β-catenin pathway is complex and summarized in Fig. 5. Regulation of the expression of components of the Wnt/β-catenin pathway by microRNAs, newly discovered RNA sequences that modify gene expression profiles, has not only revealed increased complexity of β-catenin but enabled identification of increased crosstalk between Wnt/β-catenin signaling and other pathways. Accumulating data of microRNAs will likely identify even greater complexities. In conclusion, we believe that an improved understanding of the basic genetics and biology of β-catenin signaling will provide insights into the development of novel chemopreventive and therapeutic strategies for human cancers.

## Competing interests

The authors declare that they have no competing interests.

## Authors' contributions

KH, JXZ, LH and YPY drafted and wrote the manuscript. TJ and PYP revised the manuscript critically for important and intellectual content. CSK contributed to the writing of the manuscript and supervised the project. All authors read and approved the final manuscript.
